# Detection of Sulfate-Reducing Bacteria as an Indicator for Successful Mitigation of Sulfide Production

**DOI:** 10.1128/AEM.01748-21

**Published:** 2021-11-10

**Authors:** Avishek Dutta, Fernando Valle, Thomas Goldman, Jeffrey Keating, Ellen Burke, Nicole Williamson, Reinhard Dirmeier, Jeff S. Bowman

**Affiliations:** a Integrative Oceanography Division, Scripps Institution of Oceanography, UC San Diego, La Jolla, California, USA; b BP Biosciences Center, San Diego, California, USA; c Center for Microbiome Innovation, UC San Diego, La Jolla, California, USA; University of Manchester

**Keywords:** biofilms, mitigation, potential indicator, sulfate-reducing bacteria

## Abstract

Sulfate-reducing bacteria (SRBs) are one of the main sources of biogenic H_2_S generation in oil reservoirs. Excess H_2_S production in these systems leads to oil biosouring, which causes operational risks and health hazards and can increase the cost of refining crude oil. Nitrate salts are often added to the system to suppress sulfidogenesis. Because SRB populations can persist in biofilms even after nitrate treatment, identifying shifts in the sessile community is crucial for successful mitigation. However, sampling the sessile community is hampered by its inaccessibility. Here, we use the results of a long-term (148 days) *ex situ* experiment to identify particular sessile community members from observations of the sample waste stream. Microbial community structure was determined for 731 samples across 20 bioreactors using 16S rRNA gene sequencing. By associating microbial community structure with specific steps in the mitigation process, we could distinguish between taxa associated with H_2_S production and mitigation. After initiation of nitrate treatment, certain SRB populations increased in the planktonic community during critical time points, indicating the dissociation of SRBs from the biofilm. Predicted relative abundances of the dissimilatory sulfate reduction pathway also increased during the critical time points. Here, by analyzing the planktonic community structure, we describe a general method that uses high-throughput amplicon sequencing, metabolic inferences, and cell abundance data to identify successful biofilm mitigation. We anticipate that our approach is also applicable to other systems where biofilms must be mitigated but cannot be sampled easily.

**IMPORTANCE** Microbial biofilms are commonly present in many industrial processes and can negatively impact performance and safety. Within the oil industry, subterranean biofilms cause biosouring with implications for oil quality, cost, occupational health, and the environment. Because these biofilms cannot be sampled directly, methods are needed to indirectly assess the success of mitigation measures. This study demonstrates how the planktonic microbial community can be used to assess the dissociation of sulfate-reducing bacterium (SRB)-containing biofilms. We found that an increase in the abundance of a specific SRB population in the effluent after nitrate treatment can be used as a potential indicator for the successful mitigation of biofilm-forming SRBs. Moreover, a method for determining critical time points for detecting potential indicators is suggested. This study expands our knowledge of improving mitigation strategies for biosouring and could have broader implications in other systems where biofilms lead to adverse consequences.

## INTRODUCTION

Biofilm formation is a common occurrence in many industrial processes. Biofilms can reduce efficiency and otherwise adversely impact the performance of various industrial operations (1). One example is the oil industry, which experiences considerable economic losses due to the impact of biofilm formation in pipelines, reservoirs, and other associated infrastructure (2–4). Of particular concern is oil reservoir souring, which is associated with H_2_S production by sulfate-reducing biofilms (4). Souring is a common occurrence during secondary oil recovery. During secondary recovery, the injection of seawater into an oil reservoir introduces significant amounts of the common seawater anion sulfate. These sulfate ions are utilized by sulfate-reducing bacteria (SRBs) and archaea (SRAs) to generate energy and lead to H_2_S formation, causing biosouring in the system. The production of H_2_S adversely affects downstream processes, influences the cost of oil production as well as the value of the produced oil itself, and significantly increases the health and safety risks to the workers involved in the production processes (5).

Injection of nitrate salts in oil reservoirs is one of the most effective ways for mitigate biosouring (5–9). In previous work (10), we observed prominent microbial community shifts when nitrate salts were added to a microcosm representation of a biosouring system. The addition of nitrate salts to a souring system facilitates the growth of heterotrophic nitrate-reducing bacteria (hNRBs). The presence of hNRBs lowers the pool of electron donors in the system, and hNRBs outcompete SRBs for these resources (5). Moreover, the growth of nitrate-reducing sulfur-oxidizing bacteria (NR-SOBs) is also stimulated in these systems upon nitrate salt addition (5). NR-SOBs utilize nitrate and help in reducing the sulfide pool in the system by oxidizing sulfide. Additionally, bacterial nitrate reduction also leads to the formation of nitrite in the system, which has been observed to inhibit further sulfate reduction (5, 11).

SRBs exist both in a planktonic state and in a sessile state as biofilms (12, 13), and it is generally accepted that biofilms are the main driver of oil field biosouring. In previous work (10), we observed shifts in the structure of the planktonic community after successful H_2_S suppression with nitrate addition in *ex situ* experiments. However, in that work, the state of the sessile community and potential elimination of biofilm-forming SRBs from the system during and after nitrate treatment was not known. This problem directly translates to the situation in oil fields, where the planktonic community is sampled easily when injected water exits the system, but the biofilm community is inaccessible. Moreover, some SRBs are known to shift from a sulfidogenic lifestyle to a fermentative lifestyle with changing substrate availability (14). Some SRBs can also switch from sulfate reduction to nitrate reduction when nitrate is available in the system (5). This metabolic plasticity suggests that they can persist in the oil fields even after the community has stabilized in response to nitrate addition. This persistence can lead to faster rebound sulfidogenesis when nitrate addition is halted. It is important to understand whether nitrate treatment has successfully affected the biofilm-forming SRBs in the system to maximize the effectiveness of mitigation.

This study describes a method for identifying indicators of successful biofilm intervention, even when the biofilm-forming locations are inaccessible, via signals present in the planktonic community. A long-term (148 days) *ex situ* experiment was conducted to replicate the microbial succession that occurs during and after the addition of nitrate. We used 16S rRNA gene amplicon sequencing, metabolic inference, and flow cytometry to identify signals of the putatively sessile community in the effluent stream. From this study, we gained an improved understanding of microbial dynamics during biosouring and its mitigation (by nitrate treatment), which may have broader implications in other systems (e.g., petroleum refining, paper industries, pipelines, and related systems), where biofilms can cause significant economic losses and reliable indicators of their removal are limited.

## RESULTS

### Shifts in diversity indices across sessile and effluent communities.

Twenty up-flow bioreactors were used to understand the shift in microbial diversity across biosouring and mitigation phases. Out of 20 bioreactors, nitrate salts were added to 10 columns (referred to as treated columns) to suppress sulfidogenesis, whereas the other 10 columns were used as controls where no nitrate salts were added (referred to as nontreated columns). Three main phases were observed in the treated columns, *viz*, sulfidogenesis (S), mitigation (M), and rebound sulfidogenesis (R). M phase was achieved when nitrate salts were added to the system to suppress sulfidogenesis, whereas the R phase was achieved when the nitrate treatment was stopped. A transition to the mitigation phase (TM) between sulfidogenesis and mitigation was determined where the sulfide concentration was >1 mM even after the nitrate treatment. Effluent samples were collected from each column over different time points, and microbial diversities of the samples were determined to explore the shift in community structure across different time points and phases (Table 1). Nineteen columns were sacrificed at different time points, and the sessile communities from three different sections (top, middle, and bottom) of the columns were harvested under anaerobic conditions to understand the microbial diversity of the stationary phase (Table 1).

**TABLE 1:** Details of columns showing the duration of transition phases and critical time points

Column name	No. of samples (with sessile)	No. of samples (without sessile)	Column sacrifice stage	Treatment	Temp (°C)	Sessile community harvest time points^*a*^	Transition to mitigation phase (TM phase)^*b*^	Time point during high SRB abundance (CT)^*b*^
Column 1	47	44	Rebound	Treated	30	4th	NA-ND (>1 mM sulfide)	NB
Column 2	47	44	Sulfidogenesis	Not treated	30	4th		
Column 3	38	35	Mitigation	Treated	30	3rd	NA-NC (>1 mM sulfide)	NB
Column 4	27	24	Mitigation	Treated	30	2nd	NA-NF(>1 mM sulfide)	NB and NC
Column 5	27	24	Sulfidogenesis	Not treated	30	2nd		
Column 6	25	22	Mitigation	Treated	30	1st	NA-NC (>1 mM sulfide)	NB
Column 7	25	22	Sulfidogenesis	Not treated	19	1st		
Column 8	25	22	Sulfidogenesis	Not treated	30	1st		
Column 9	25	22	Sulfidogenesis	Not treated	19	1st		
Column 10	25	22	Mitigation	Treated	19	1st	NA-NE (>1 mM sulfide)	NB and NC
Column 11	38	35	Sulfidogenesis	Not treated	30	3rd		
Column 12	47	44	Sulfidogenesis	Not treated	30	4th		
Column 13	38	35	Mitigation	Treated	30	3rd	NA-NC (>1 mM sulfide)	NB and NC
Column 14	27	24	Mitigation	Treated	19	2nd	NA-NG (>1 mM sulfide)	NB, NC, and NE
Column 15	47	44	Rebound	Treated	30	4th	NA-NC (>1 mM sulfide)	NB and NC
Column 16	47	44	Sulfidogenesis	Not treated	30	4th		
Column 17	38	35	Sulfidogenesis	Not treated	30	3rd		
Column 18	47	44	Rebound	Treated	30	4th	NA-NC (>1 mM sulfide)	NA and NB
Column 19	47	44	Rebound	Treated	30	4th	NA-NE (>0.94 mM sulfide)	NB and NC
Column 20^*c*^	44	44	Not harvested	Not treated	30			

a Dates for the four harvests are as follows (mo/day/yr): 1st, 08/23/2019; 2nd, 08/28/2019; 3rd, 10/07/2019; and 4th, 11/11/2019.

b Time points for TM phase and CT are as follows: NA, 07/29/2019; NB, 07/31/2019; NC, 08/02/2019; ND, 08/05/2019; NE, 08/07/2019; NF, 08/09/2019; and NG, 08/12/2019.

c No sessile samples were harvested for column 20.

**FIG 1:**
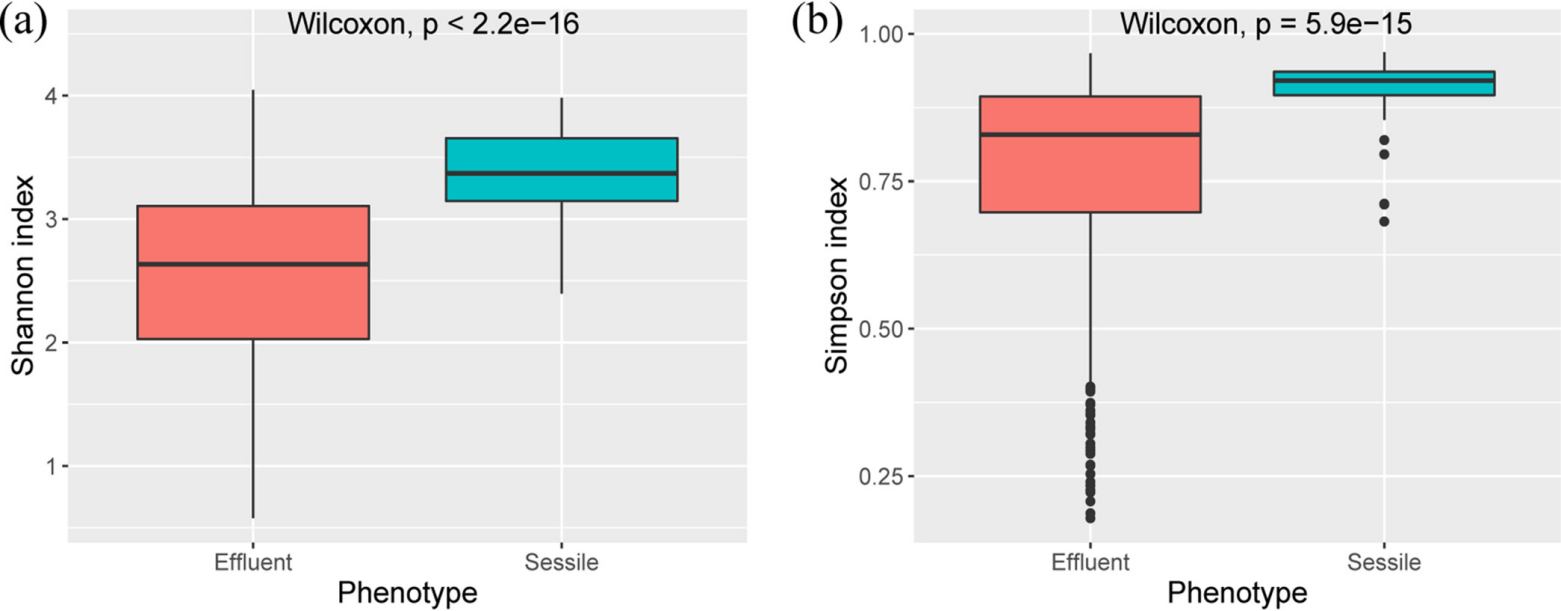
Box plot of Shannon’s indices (a) and Simpson’s indices (b) for effluent and sessile communities of treated and nontreated columns.

**FIG 2:**
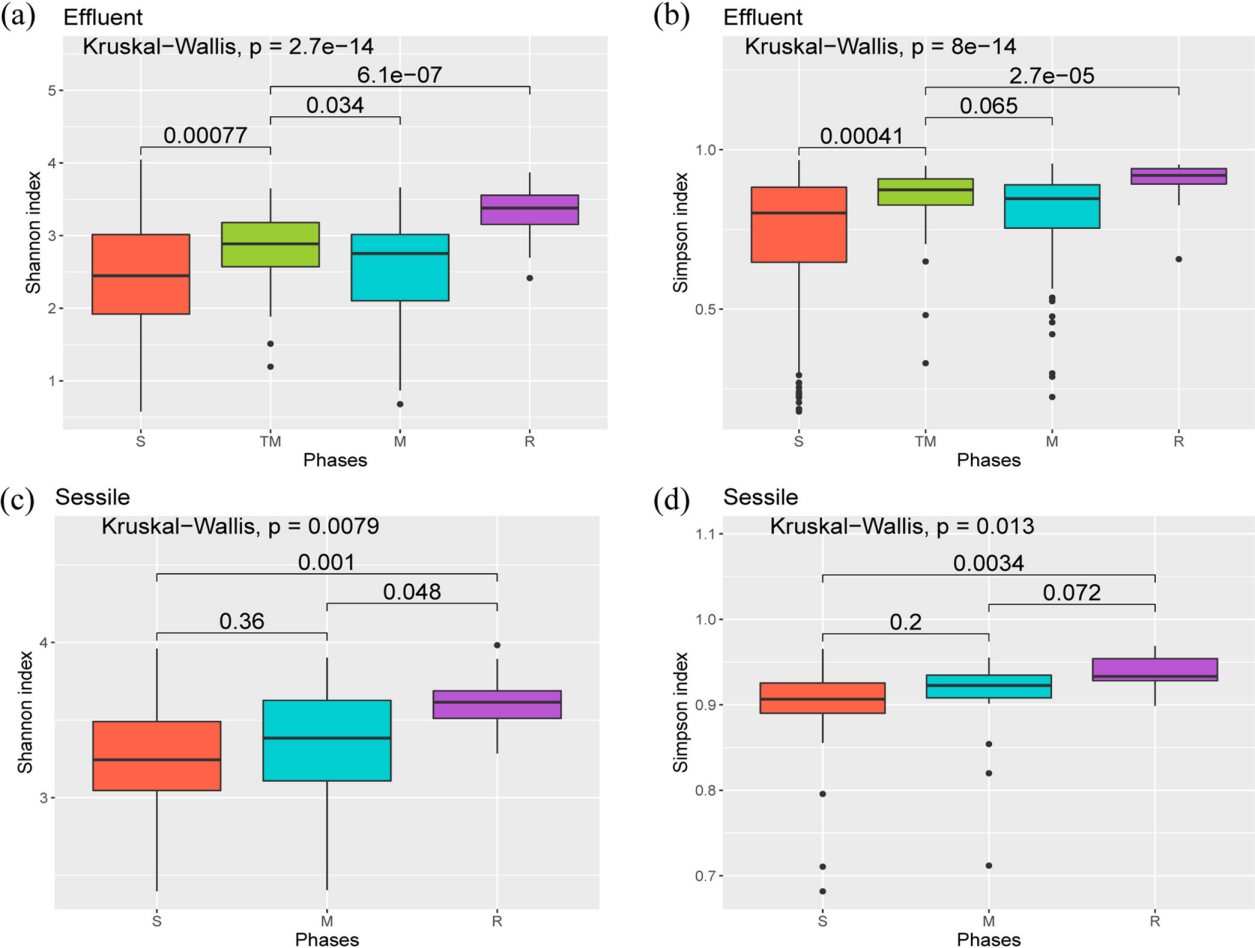
Box plot of Shannon’s indices (a) and Simpson’s indices (b) for effluent communities across different phases of treated and nontreated columns. Box plot of Shannon’s indices (c) and Simpson’s indices (d) for sessile communities across different phases of treated and nontreated columns. A pairwise comparison for significance was conducted using the Wilcoxon test.

Shannon’s diversity index, which evaluates species abundance and evenness, and Simpson’s index, which measures the species dominance in a community, were calculated for the samples. The differences in Shannon’s and Simpson’s indices in sessile and effluent samples were analyzed. Shannon’s and Simpson’s indices of the sessile samples were significantly higher (Wilcoxon tests, *P < *2.2 × 10^−16^ for Shannon’s diversity index and *P *= 5.9 × 10^−15^ for Simpson’s index) than those of the effluent samples across all the phases (Fig. 1a and b). The Shannon index showed that the microbial diversity of the TM phase was significantly higher than the diversity of S (*P *= 0.00077) and M (*P *= 0.034) phase effluent samples (Fig. 2a). The Shannon index of the effluent samples from the R phase (Fig. 2a) was found to be significantly higher than that from S, TM, and M phases (R versus S, *P *= 4.2 × 10^−13^; R versus TM, *P *= 6.1 × 10^−7^; R versus M, *P *=* *6.8 × 10^−12^). The Simpson’s microbial diversity for effluent samples across different phases displayed a similar pattern (R versus S, *P *= 7.8 × 10^−12^; R versus TM, *P *= 2.7 × 10^−5^; R versus M, *P *=* *2.1 × 10^−9^) (Fig. 2b). For sessile samples, the shift in the Shannon’s diversity index across phases also displayed a similar pattern, where the microbial diversity for sessile samples from the R phase was significantly higher than that for S and M phase sessile samples (R versus S, *P* = 0.001; R versus M, *P* = 0.048) (Fig. 2c). Microbial diversities for sessile samples from S and M phases were not significantly different (*P *= 0.36 for Shannon’s diversity index and *P *= 0.2 for Simpson’s diversity index) (Fig. 2c and d).

### Similarities and dissimilarities in microbial community structures across different phases of sessile and effluent samples.

**FIG 3:**
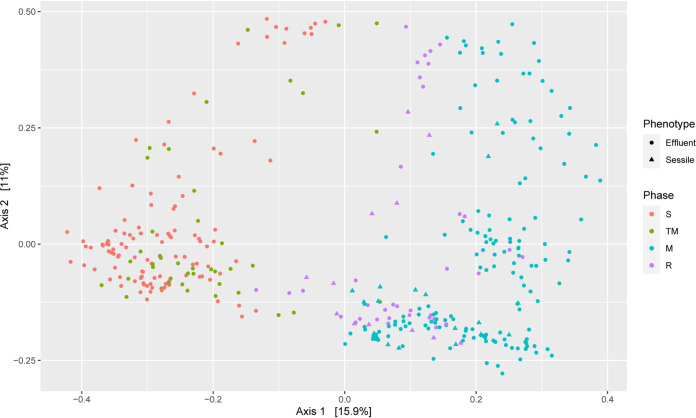
Principal coordinates analysis (PCoA) of Bray-Curtis dissimilatory based on the relative abundances of all unique sequences across different phases of treated columns.

A principal-coordinate analysis (PCoA) based on the Bray-Curtis dissimilarity of all the samples from the treated columns was done to compare the diversity across different phases. A clear separation of samples from S and M phases was observed (Fig. 3). Samples from the TM phase clustered with the S phase samples, whereas samples from the R phase were closer to the M phase samples.

**FIG 4:**
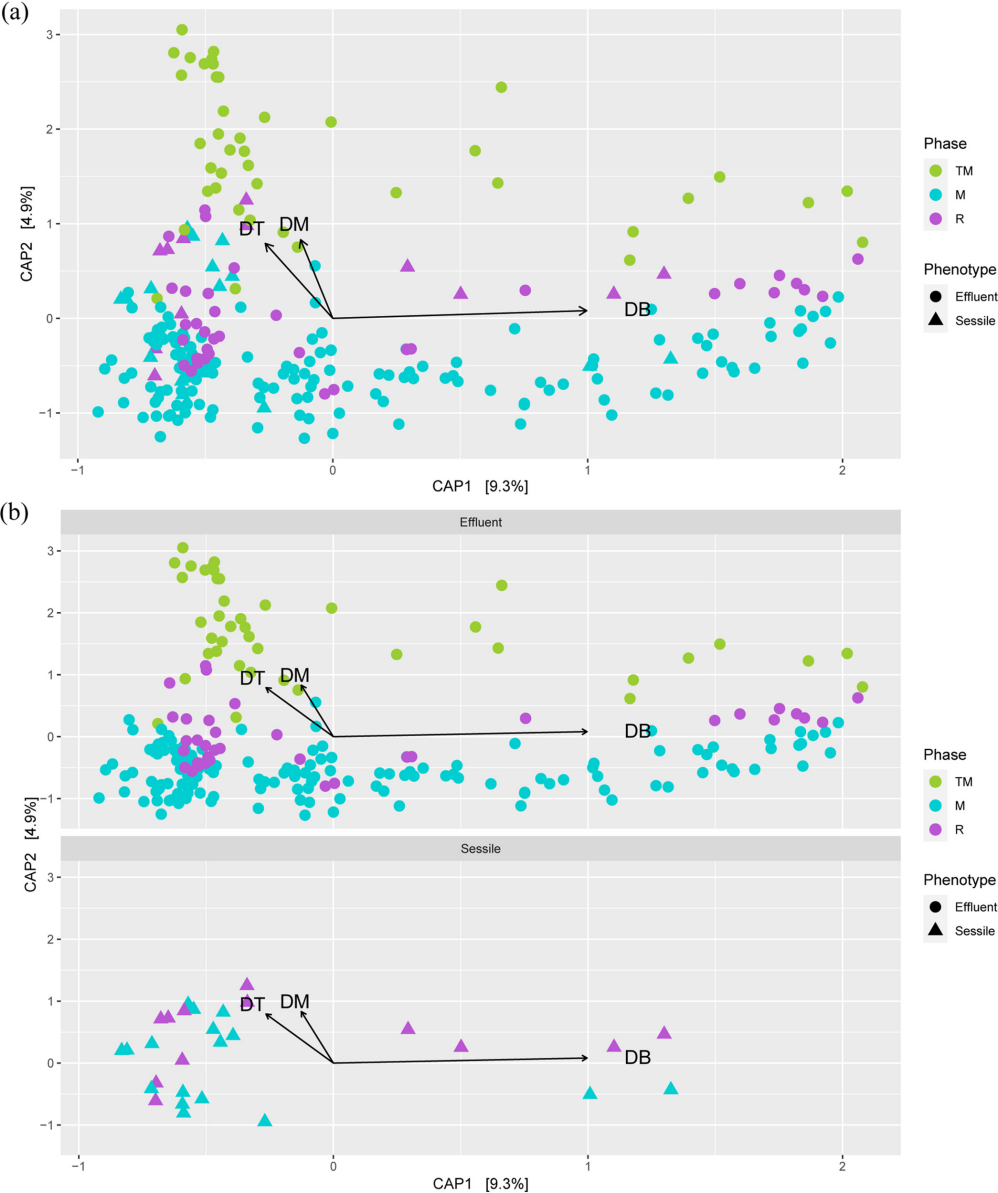
Canonical analysis of principal coordinates (CAP) of Bray-Curtis dissimilatory based on relative abundance of all unique sequences from TM, M, and R phases constraining three most abundant SRBs in the treated column (a). (b) Facet plots of the same CAP showing separate plots for sessile and effluent samples. DT, Desulfobacula toluolica Tol2; DB, Desulfarculus baarsii DSM 2075; DM, Desulfococcus multivorans.

A canonical analysis of principal coordinates (CAP) was conducted based on samples from TM, R, and M phases of treated columns constraining the top three most abundant SRBs (Fig. 4a and b). The closest completed genomes of the three most abundant SRBs were found to be Desulfarculus baarsii DSM 2075, Desulfobacula toluolica Tol2, and Desulfococcus multivorans. A CAP analysis was done to understand the grouping of samples based on the abundances of the top three most abundant SRBs across all the samples. Distinct clusters for effluent samples from the M and TM phases were observed. The samples from the M phase were observed in quadrants III and IV of the CAP plots, whereas samples from the TM phase were observed mainly in quadrants I and II of the CAP plots. Interestingly, most of the samples from the sessile communities clustered with samples from TM phases (in quadrants I and II), and the vectors for the top three SRBs were directed toward the same quadrants, indicating a higher presence of these SRBs in TM and sessile samples than in the samples from the M phase.

**FIG 5:**
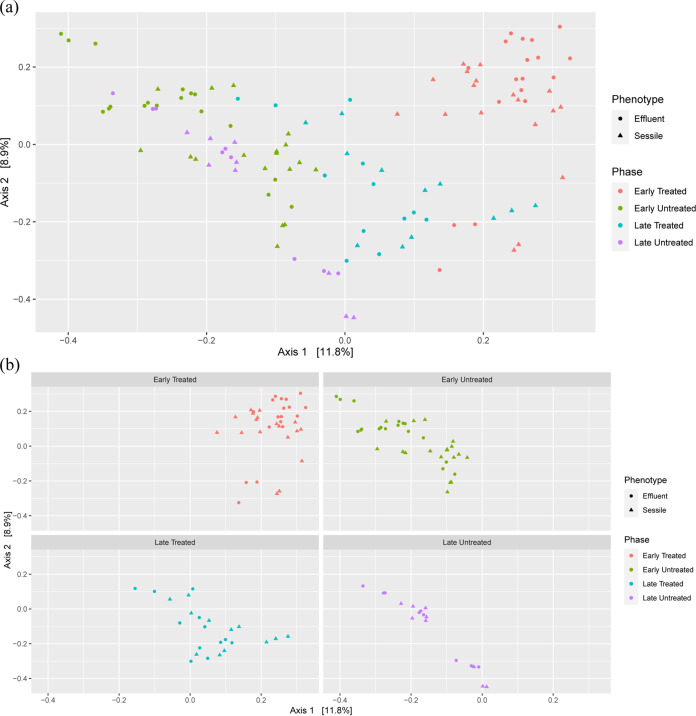
Principal coordinates analysis (PCoA) of Bray-Curtis dissimilatory based on the relative abundances of all unique sequences across sessile samples and three effluent time points of each column (before the columns were sacrificed for sessile community harvesting). (a) PCoA plot showing observations from all the time periods. (b) Facet plots of the same PCoA showing separate plots for different time periods. The PCoA plot has been segregated based on late- and early-harvesting time periods of treated and nontreated columns. Early harvesting represents columns harvested during the 1st, 2nd, and 3rd harvest, whereas late harvesting represents columns harvested during the 4th harvest. Details of columns and time periods are present in Table 1.

A PCoA of Bray-Curtis dissimilarity across sessile and selected effluent samples (samples from three time points before the columns were sacrificed) displayed clear patterns (Fig. 5a and b). For the treated columns, sessile samples were harvested during two phases (M and R), covering four time points (Table 1). The 1st, 2nd, and 3rd harvest were conducted during the M phase and considered early treated, whereas the 4th harvest was conducted during the R phase and considered late treated. The sessile samples harvested during the M (early treated) and R phases (late treated) of the treated columns showed distinct clustering. Most of the samples from the early-treated phase clustered in quadrant I, whereas samples from the late-treated phase clustered in quadrant IV. The harvesting time points for early and late periods for treated and nontreated columns were kept the same to compare the differences in treated and nontreated columns (Table 1). In nontreated columns, the sessile and the effluent samples obtained from the 1st, 2nd, and 3rd harvest (Table 1) (referred to as early untreated in the PCoA plots of Fig. 5a and b) grouped closely with the sessile and effluent samples from the 4th harvest (late untreated) in quadrants II and III. It was interesting to note that irrespective of the phases and column treatment (whether treated or nontreated), sessile samples grouped closely with the effluent samples for a particular phase. The overall comparison showed that the samples from the treated and nontreated columns (irrespective of whether the samples are effluent or sessile) were grouped separately. The samples from the nontreated columns were clustered in quadrants II and III, whereas the samples from the treated columns clustered in quadrants I and IV.

### SRBs as a potential indicator for successful mitigation.

**FIG 6:**
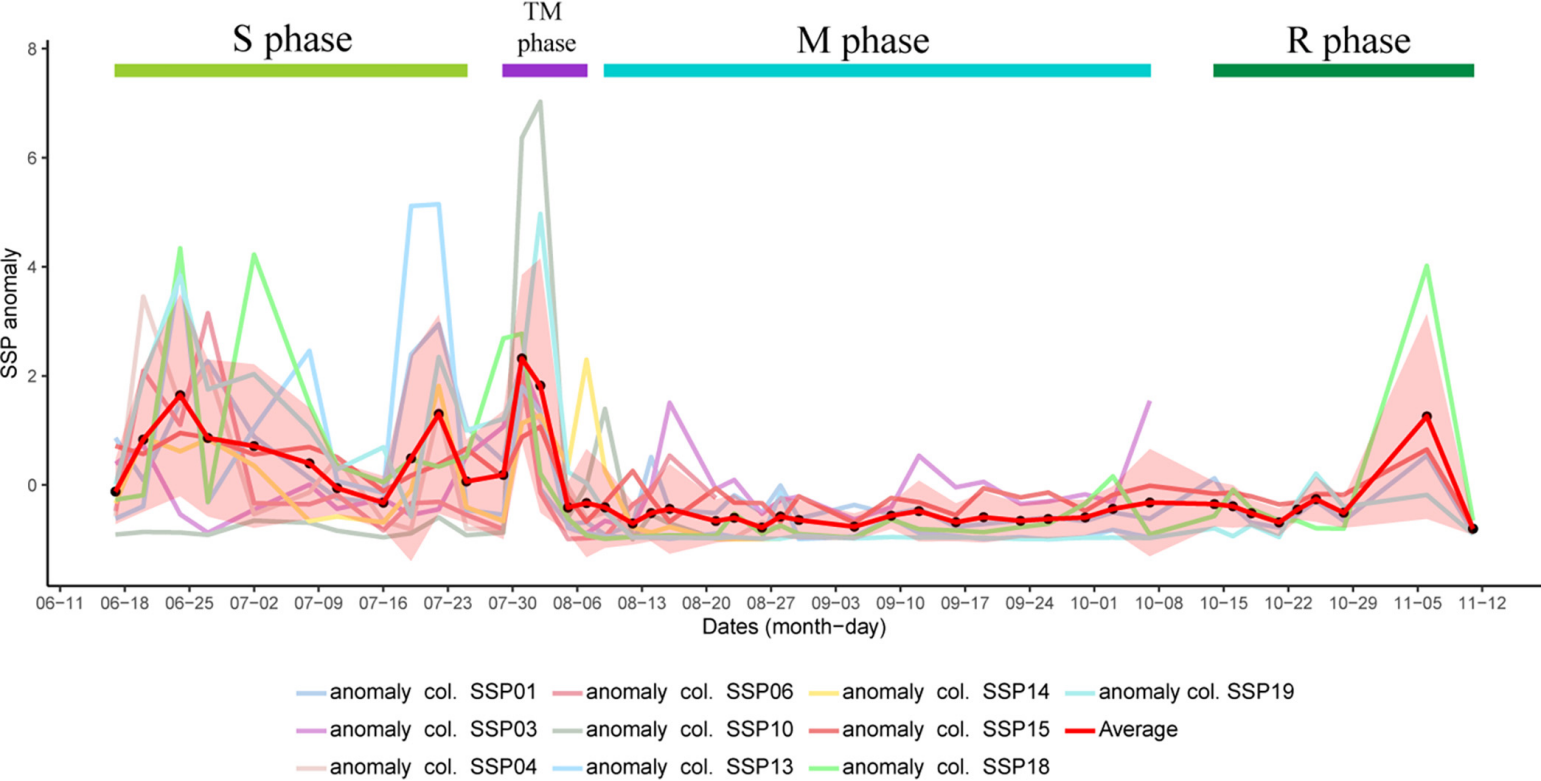
Anomaly plots based on the relative abundance of specific SRB population (SSP) across different time points of the treated columns. The closest completed genome of SSPs for column 1, 4, 10, 13, 14, 18, and 19 is Desulfobacula toluolica Tol2; for column 2 and 15 is Desulfarculus baarsii DSM 2075; and for column 6 and 14 is Desulfococcus multivorans. Column 14 had two SSPs. The shaded region in the plot depicts the standard deviation for the average anomaly plot. Critical time points for each column are present in Table 1.

While the microbial community shift from S to M phase (during the TM phase) was analyzed, a sudden increase in SRB populations was observed in all the treated columns. This increase in the relative abundance of a specific SRB population (SSP) across different columns was further analyzed. The abundance of SSP was obtained by adding up the abundances of all the unique sequences assigned to a particular closest completed genome of an SRB. Anomalies of the relative abundance of SSPs were ascertained across different time points to understand the shift in SSP abundance as a proportion of the total community across different time points (Fig. 6). SSPs varied across different phases and columns. It was found that the relative abundance of the SSP increased in the TM phase. Desulfobacula toluolica Tol2 was found to be the SSP in columns 1, 4, 10, 14, 18, and 19, whereas Desulfarculus baarsii DSM 2075 was observed to be the SSP in columns 3 and 15. Desulfococcus multivorans was found to be the SSP in columns 6 and 14. It was interesting to note that in column 14, both Desulfobacula toluolica Tol2 and Desulfococcus multivorans were present in higher abundances in effluent samples of the TM phase.

It was found that specific time points from the TM phase displayed a higher relative abundance of the SSPs compared with the others. These time points varied across columns, as did the length of the TM phase (Table 1, Fig. 6). The time points during the TM phase, where relative SSP abundances were found to be higher than the average relative SSP abundance in the TM phase, were considered to be critical time points. Critical time points are the time points where the potential indicators (SSPs in this study) are detected in high abundances. Two days after the first application of nitrate treatment (time point NB) was found to be the critical time point for all the columns, whereas time point NB and 4 days after the first application of nitrate (time point NC) were found to be the critical time points for 6 of the 10 treated columns. It was interesting to note that for 9 out of 10 treated columns, the first time point after nitrate treatment (time point NA) did not fall under the criterion of a critical time point.

**FIG 7:**
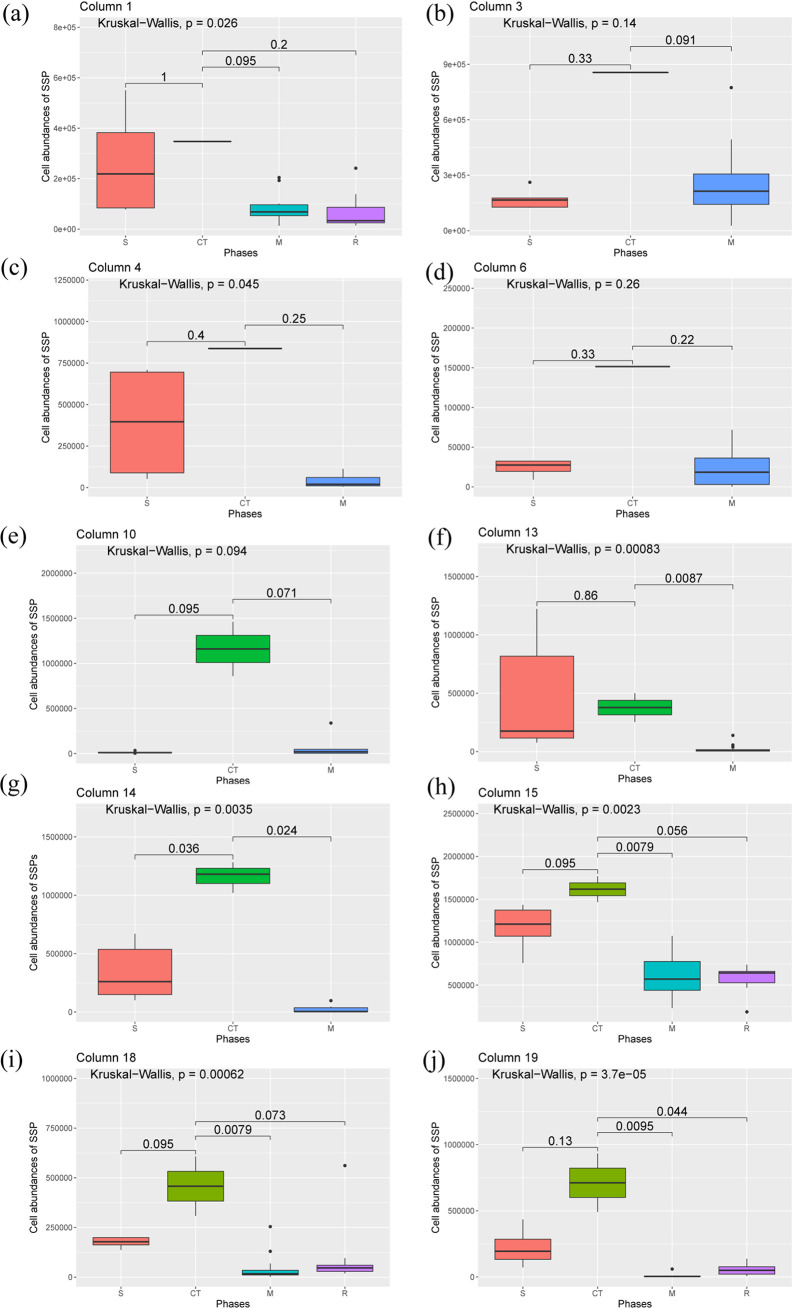
Box plots showing the distribution of cell abundances of specific SRB population (SSP) across different phases of column 1 (a), column 3 (b), column 4 (c), column 6 (d), column 10 (e), column 13 (f), column 14 (g), column 15 (h), column 18 (i), and column 19 (j). S, CT, M, and R denote effluent samples from sulfidogenesis, critical time point, mitigation, and rebound-sulfidogenesis phases, respectively. The Kruskal-Wallis test was used to understand whether the overall changes in cell abundances of SSPs across different phases are significant. The values mentioned for pairwise comparisons of cell abundances between phases are the *P* values from the Wilcoxon test.

Three different SRB populations, *viz*, Desulfobacula toluolica Tol2, Desulfarculus baarsii DSM 2075, and Desulfococcus multivorans, were found to be possible indicators of successful mitigation in this experimental setup and were specific to different columns. Box and whisker plots were drawn to understand the changes in cell abundance of the SSPs across different phases of the treated columns (Fig. 7). It is evident that for a particular column, the cell abundance of SSPs during critical time points (if there was only one critical time point) or the median of cell abundances of SSPs during critical time points (if there is more than one critical time point) is always higher than the median of cell abundances of SSPs in other phases of the effluent samples. A Kruskal-Wallis test for significance revealed that the overall changes in the SSP abundances across different phases were significant (*P < *0.05) for seven treated columns.

### Shift in predicted dissimilatory sulfate reduction (DSR) pathway abundance across different columns.

**FIG 8:**
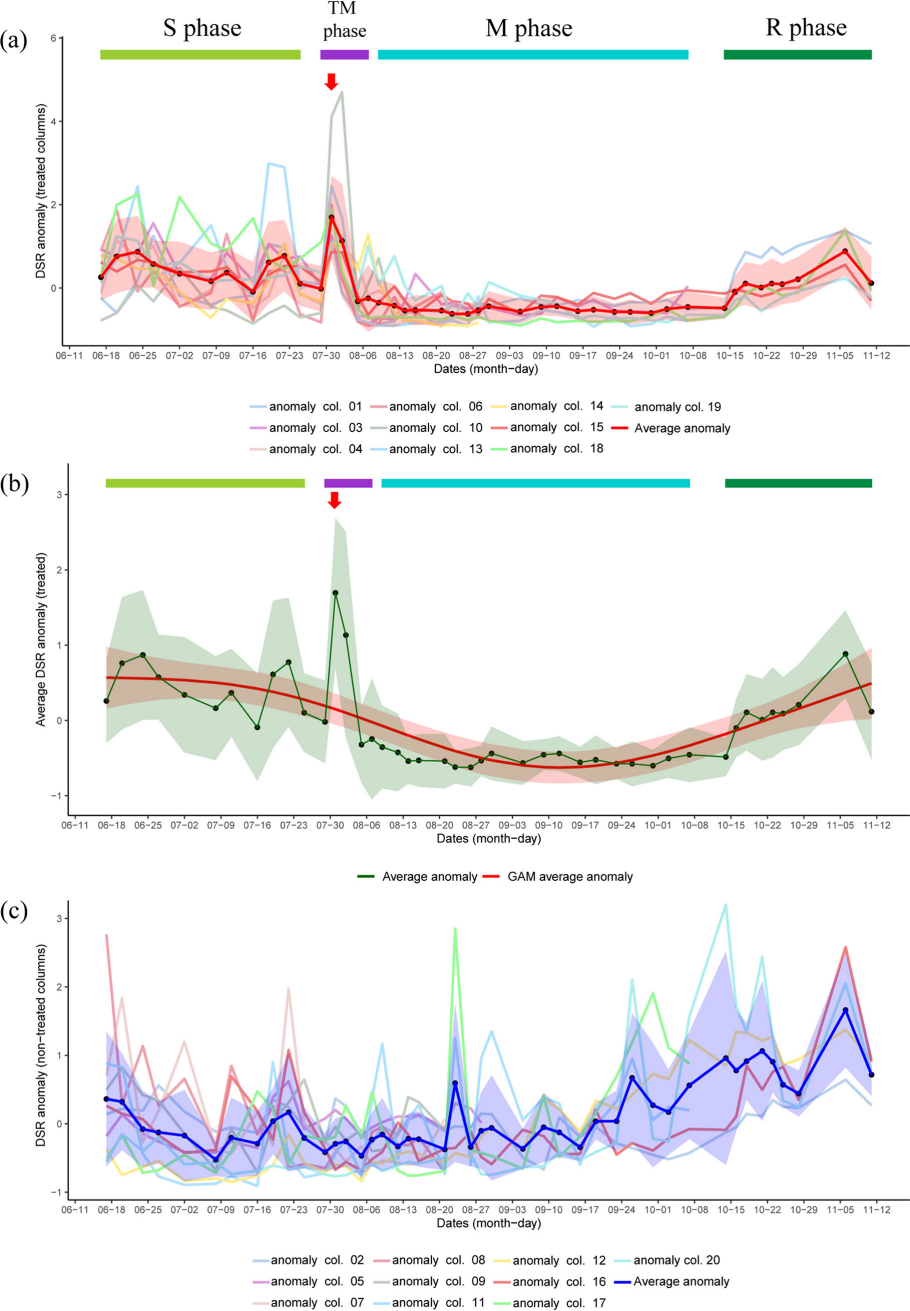
Shift in dissimilatory sulfate reduction pathway (DSR) abundance across different time points in treated and nontreated columns. (a) Anomalies of DSR pathway abundances in treated columns along with the average anomaly for each time point. Initiation of the TM phase indicates initiation of nitrate treatment, and the red arrow represents time point NB. (b) Average anomaly of DSR with line smoothing using a generalized additive model (GAM) in treated columns. Initiation of TM phase indicates initiation of nitrate treatment, and the red arrow represents time point NB. (c) Anomalies of DSR pathway abundances in nontreated columns along with average anomaly for each time point. Shaded regions in the plots indicate standard deviations for average anomaly plots, whereas the shaded region for the GAM plot indicates ± 2 standard error.

Anomalies of the relative abundance of dissimilatory sulfate reduction I (to hydrogen sulfide) (DSR) as predicted from paprica (15) were analyzed for both the treated and nontreated columns (Fig. 8). This analysis was done to obtain a unified indicator that could be used to understand the increase in SRB abundance in the effluent during critical time points. Overall, the results suggest that higher values of the anomaly were observed in the S phase than those in the M phase (Fig. 8a). The shift in average anomaly (calculated for each time point) also showed similar results (Fig. 8a). Interestingly, the average anomaly peaked during time point NB, which was determined to be the critical time point for all the treated columns. The second-highest average anomaly value was obtained for time point NC, which was found to be the critical time point for six treated columns. A generalized additive model (GAM) was created to obtain an overall trend of the average anomaly across different time points (Fig. 8b). The trend from the GAM analysis indicated that the anomaly values started decreasing as nitrate amendment was initiated (during the M phase) and then started increasing when nitrate addition in the system was stopped (during the R phase). It was interesting to note that during critical time points, the trend obtained from the GAM analysis suggests a decrease in the anomaly.

Anomalies of relative DSR abundance were also calculated for the nontreated columns to analyze and compare the shift across different time points (Fig. 8c). The results indicated no consistent trend in the DSR relative abundances in the nontreated columns compared with those in the treated column. The shift in average anomalies of DSR relative abundances for nontreated columns was not similar to the shift as observed in the treated columns.

## DISCUSSION

Biofilms are found in many industrial processes and can adversely affect the performance and safety of the operations. Oil industries are no exception because biofilms are known to cause biofouling and corrosion. Furthermore, biofilm-forming SRBs in oil reservoirs lead to biosouring, which negatively impacts oil quality, cost, and occupational health. Since these subterranean biofilms are hard to access and sample, it is difficult to know whether successful mitigation of biosouring has affected the sessile SRB populations. Moreover, the scavenging and transformation of H_2_S in the oil reservoirs make it hard to assess the mitigation efficiencies from the surface. The present study describes a method that uses high-throughput 16S rRNA gene sequencing, metabolic inferences from paprica, and cell count from flow cytometry to detect a sudden increase in SRB populations in the planktonic community during mitigation of biosouring via nitrate treatment. This sudden increase in SRB populations in the planktonic community (referred to as effluent community in the manuscript) can be used as an indicator for the successful mitigation of biosouring and associated biofilms. The microbial community associated with different phases in effluent and sessile samples explored in this study gives us a unique window for identifying bioindicators for successful mitigation. Moreover, this study broadens our knowledge of the microbial community shifts associated with different phases of biosouring and mitigation treatments.

The alpha diversity pattern (based on Shannon’s diversity index) among different phases of the effluent community suggests that the microbial diversity of TM phase was higher than that of the S and M phases. This result indicates that the richness and evenness of the effluent microbial community during the TM phase are higher than those during the S and M phases of the effluent samples. This sudden increase in the richness of microbial diversity after the addition of nitrate salts (during the TM phase) in the system followed by a decrease in diversity and richness (during the M phase) may be attributed to the dissociation of microbial populations from the biofilms. This dissociation may be triggered by several factors, such as shifts in nutrient availability and/or replacements of microbial populations in the biofilm. The alpha diversity pattern for the sessile microbial community also supports the previous hypothesis. Similarities in changes in diversity indices in both the effluent and the sessile communities were observed. This finding indicates that the shift in microbial community diversity may be linked; changes in the microbial diversity of the sessile community may influence the changes in the effluent community and vice versa. No significant differences in alpha diversity patterns of the sessile samples from S and M phases were observed. This result suggests that either a shift in the metabolic lifestyle of microbes from the S phase has led to their persistence in the M phase or replacements of microbes in the sessile community have helped to maintain a similar diversity, richness, and evenness in the samples. Ordination analyses based on microbial community structures were conducted to further understand and support the observations from alpha diversity analyses.

The PCoA analysis based on microbial community structures of all the samples from the treated columns suggests that unique microbial communities are associated with each phase. A distinct grouping of the samples from the S and M phases suggests that nitrate treatment facilitates the shift in microbial community structure. The close grouping of samples from the S and TM phases gives us a sense that each phase influences the subsequent phase but that with time distinct microbial communities are established in different phases. This hypothesis is further supported by the close grouping of samples from the M and R phases. A CAP analysis based on constraining the top three SRBs (Desulfarculus baarsii DSM 2075, Desulfobacula toluolica Tol2, and Desulfococcus multivorans) supported the hypothesis obtained from alpha diversity analyses (Fig. 4a and b). The close grouping of the TM phase effluent samples with the sessile samples from most of the columns indicates the presence of those three SRBs in both the sessile samples and the TM phase effluent samples. Moreover, this finding also indicates that dissociation of SRBs from the biofilms may have led to the increase in SRB abundance in the effluents, even after initiation of the nitrate treatment.

To understand the relationships between sessile and effluent communities across treated and nontreated columns, a PCoA based on the Bray-Curtis dissimilarity of all the bacterial populations from sessile and three effluent time points of each column was conducted. As shown in Fig. 5, the sessile samples and the effluent samples for a particular phase were grouped closely. This result indicates that sessile sample communities vary with the phases and the microbial community structures of sessile samples are similar to those of the effluent samples for a particular phase/time point. For nontreated columns, the shift in bacterial community structure for both sessile and effluent samples did not vary much during early and late harvesting periods. In contrast, the community structure of sessile and effluent samples for treated columns during the M phase (early harvesting), and the R phase (late harvesting) were different. This result suggests that nitrate treatment has an impact on both sessile and effluent communities.

Similar microbial community shifts were observed in sessile and effluent communities across phases. Since biofilms are often hard to assess due to a lack of accessibility for sampling, this study used the effluent microbial community structure to understand the changes in sessile communities. Our results suggest that the nitrate treatment facilitates the dispersal of sessile community members from the biofilm formed during the sulfidogenic phase. There are at least two possible mechanisms that can accelerate the process of biofilm dispersal in the system after the addition of nitrate. First, the formation of nitric oxide (NO) in the system upon the addition of nitrate may facilitate biofilm dispersal. The addition of nitrate in such systems often promotes the growth of heterotrophic denitrifiers, which produce NO as a product of denitrification. Previous studies have reported the role of NO in biofilm dispersal (16). Second, the formation of glutamate may also accelerate the process of biofilm dispersal. Glutamate is a key central metabolite at the crossroad of cellular anabolism and catabolism, as well as the starting point for anaplerosis of N-containing metabolites and for nitrogen assimilation (16). Amendment of nitrate increases the nitrogen pool in the system, thus increasing the production of glutamate. A previous study reported the role of glutamate in biofilm dispersion, where glutamate stimulates biofilm dispersion through the production of matrix-degrading enzymes (17).

Biofilm dispersal may also be triggered by nitrate reduction in the system, which is different from nitric oxide formation. One of the intermediates of nitrate reduction is nitrite, which has been observed to inhibit sulfate reduction (5, 11). The introduction of nitrate in the system enhances the growth of hNRBs and NR-SOBs, thus increasing the nitrite pool in the system. This increase in the nitrite concentration inhibits dissimilatory sulfite reductase (Dsr) of the SRBs. The inhibition of Dsr eventually cuts off the energy source of those SRBs, which depend solely on anaerobic respiration. This process may also lead to an energy-limiting condition for the SRBs in the biofilm, which may facilitate biofilm dispersal. After the biofilms are disrupted by the production of intermediates of nitrogen metabolism, new bacterial populations start to colonize the system. This recolonization in the sessile samples of the treated columns can be supported by the hypothesis made from alpha diversity analyses, where no significant differences in bacterial diversity richness and evenness were observed between sessile samples from S and M phases.

These analyses and hypotheses suggest that the colonization of new members in the sessile samples after the nitrate amendment is accompanied by biofilm dispersal. This suggestion was evident from the anomaly analyses based on relative SSP abundance. A sudden increase in the SSPs was observed within 2 to 4 days after the initiation of the nitrate treatment. The increase in SSP abundances after nitrate salt addition suggests the dispersal of SRBs from biofilms. The dispersal of SSPs from the biofilm can be used as an indicator of the successful mitigation of biosouring. It was found that Desulfobacula toluolica Tol2, Desulfarculus baarsii DSM 2075, and Desulfococcus multivorans can be used as a potential indicator for this system. Although the relative abundances of these SSPs varied across the TM phase, critical time points were identified for these systems, which varied between 2 to 4 days from the first application of nitrate. It was also interesting to note that cell abundances of the SSPs were also found to be higher during critical time points than those during S, M, and R phases (Fig. 7). Critical time points are the time points where the potential indicators are detected in high abundances. The first time point after nitrate amendment (time point NA) did not fall under the criterion of critical time points for 9 out of 10 treated columns. This finding indicates that the processes responsible for the dispersal of biofilms need some time to get established in the system after the initiation of nitrate treatment. It seems that the growth of NRBs or other bacterial populations related to nitrogen cycling in the system is a determinant of the critical time points. It appears that the time required for the stable growth of NRBs or related bacterial populations is the lag between the initiation of the TM phases and the first critical time point. This result further supports the previous hypothesis where the product of denitrification (nitric oxide) or nitrogen metabolism (glutamate) leads to the dispersal of biofilms. Since the TM phases of all the columns extended beyond the first critical time point, we conclude that the detection of potential indicators in the critical time points can also be used as an early indicator for successful mitigation.

Anomalies based on the relative abundance of the DSR pathway were analyzed for the treated columns to support the previous hypotheses (Fig. 8). The results were in line with the changes in relative and absolute abundances of SSPs observed across different phases. The increase in the average anomaly of DSR during time point NB (for the treated columns) further indicates the rise in SRB abundance in the effluent during time point NB (critical time point for all the columns). It was interesting to note that the GAM analysis suggests a decrease in the anomaly during critical time points. The spike observed from the actual data compared with the GAM analysis indicates that the increase in SRB population in the effluent during critical time points is not an expected phenomenon but was triggered by certain external changes in the environment. As the only external change during this time point was the addition of nitrate salts, we conclude that the increase in the SRB population in the effluent during the critical time points was triggered by nitrate addition. Since the growth of SRBs should not be enhanced in the presence of nitrate, dispersal of SRBs from the biofilm leads to an increase in SRB abundance in the effluent during the critical time points. Inconsistent anomalies for DSR abundance in the nontreated columns further support that the surge in DSR abundances during the critical time points in the treated column was not an experimental artifact or a random event.

**FIG 9:**
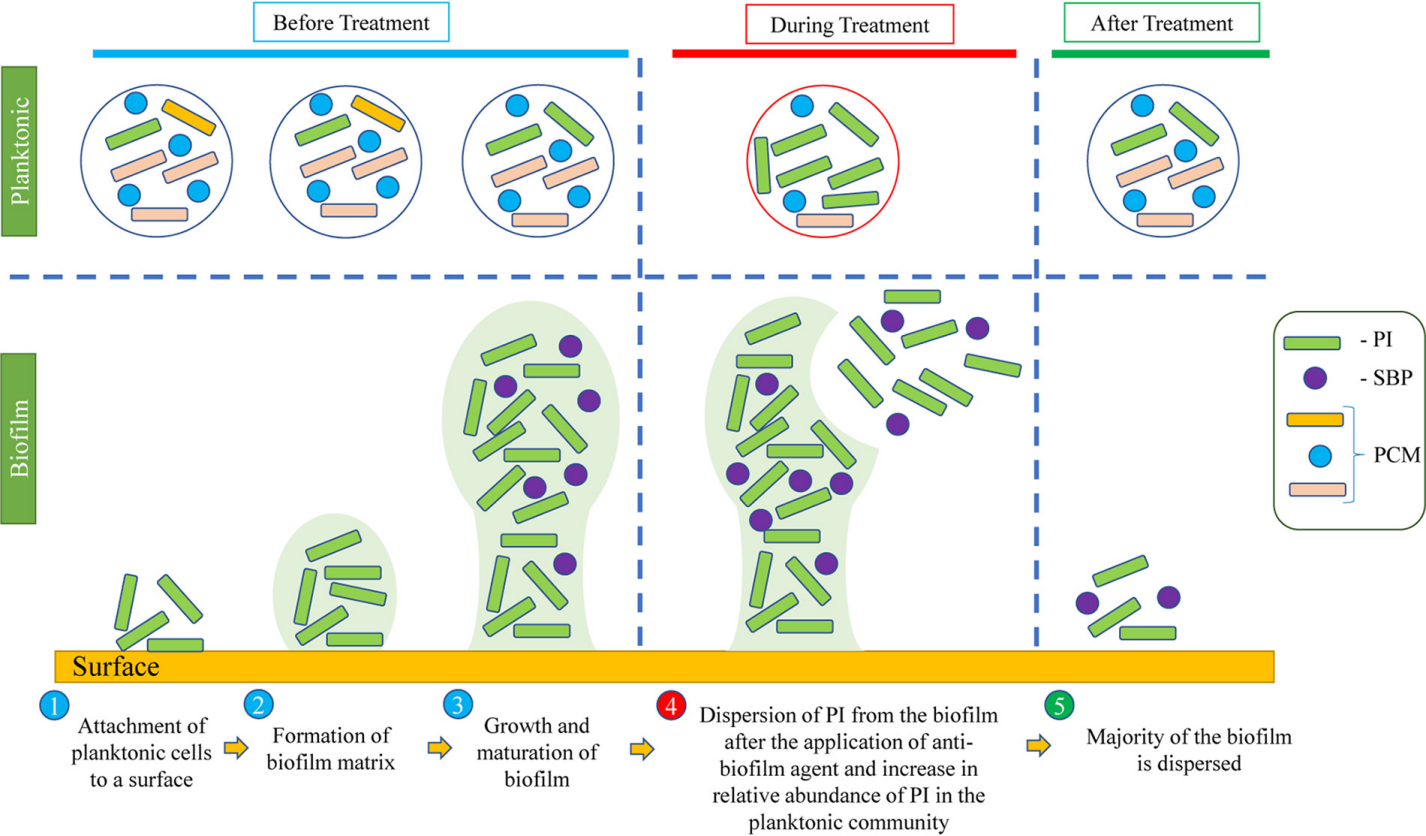
Detection of potential indicators after application of the biofilm eradication method. Here, treatment refers to the application of the biofilm eradication method. PI, potential indicator; SBP, secondary bacterial populations; PCM, planktonic community members. Critical time points are observed during stage 4.

Critical time points and potential indicators will vary from system to system, depending on environmental conditions and the volume of the system. Overall, results suggest that critical time points and potential indicators can be identified at the beginning of an oil field operation or any pilot plant study, which could further help in assessing successful mitigation and changes in sessile communities. Since actual H_2_S concentrations cannot always be detected on the surface due to scavenging in the reservoir, the planktonic microbial populations can be used as a marker for the successful mitigation of biosouring and biofilms. Although an increase in NRB populations in effluents can be used as an indicator for the successful mitigation of biosouring, it does not necessarily mean eradication of biofilm-forming SRBs from the system. SRBs can change their metabolic lifestyle (5, 10, 14) and still persist after nitrate treatments. The persistence of SRBs in biofilms can lead to faster rebound sulfidogenesis once the nitrate treatment is stopped. The proposed method gives an opportunity for understanding whether nitrate treatment has affected the biofilm-forming SRBs, which can further help to maximize the effectiveness of mitigation. This method could have broader applications beyond oil field operations. A general application of this procedure to identify successful biofilm mitigation for a process that is impeded by biofilm formation is described in Fig. 9. During normal operation, the microbial community in the effluent can be monitored by an analysis of the 16S rRNA gene using standard filtrating and sequencing methods. A “healthy” biofilm will be present minimally in the planktonic bacterial community, and regular monitoring establishes a baseline against which to detect community shifts. When a mitigation strategy is applied to disrupt the biofilm, sessile members of the biofilm community are “sloughed” into the effluent, where they can be observed. The effluent can be monitored for evidence of the biofilm, and an increase in sessile members of the microbial community indicates the successful disruption of the biofilm.

This study explored the shift in microbial community structures across different phases of biosouring and mitigation to detect specific members of the effluent microbial community, which can be used as an indicator for successful biosouring and biofilm mitigation. Of particular interest was our finding that during the TM phase, a significant part of the microbial diversity consisted of SRB. Since these SRBs were also part of the sessile microbial population, it seems that the disintegration of biofilm is contributing to the SRB population in the effluent during the TM phase. An increase in the cell abundance of a particular SRB population in the effluent after nitrate amendment could be used as a potential indicator for the successful mitigation of biosouring in oil reservoirs. Critical time points were also determined based on the abundance of potential indicators in the TM phase. In similar systems, potential indicators and critical time points could be identified for enhancing the mitigation strategies for biosouring using nitrate salts. This hypothesis was also supported by the anomaly analysis based on the relative abundance of the DSR pathway, where a sudden spike in anomaly was observed during critical time points. This study not only furthers our knowledge for improving mitigation strategies for biosouring but also could have broader implications in other systems, where biofilms lead to significant negative impacts.

## MATERIALS AND METHODS

### Experimental setup.

Twenty up-flow bioreactors were used to understand the shift in microbial diversity across different phases of biosouring and mitigation. Briefly, US Silica ASTM-graded sand, unground silica was used to pack the columns. Souring was first initiated using seawater and volatile fatty acid (VFA) flowthrough, followed by subsequent flowthrough and shut-in of yeast extract-enriched seawater culture from anoxic serum bottles. H_2_S concentrations were measured in the system using the Cline assay (19). For the H_2_S measurement, collection tubes were screwed onto the effluent line of the bioreactors for 1 hour to collect samples, and the sample aliquot was withdrawn for the Cline assay. Seawater, collected from Scripps Pier, was injected into all the 20 bioreactors at a flow rate of 1 ml hr^−1^. VFAs were added in each column to promote sulfidogenesis and mitigation. A total of 33 mM VFAs (equimolar of acetate, butyrate, formate, and propionate) was added at a flow rate of 100 μl h^−1^ leading to a column influent concentration of 3 mM when mixed with 1 ml hr^−1^ seawater. The anoxic condition was initiated using a 100% N_2_ flush to remove O_2_, followed by a 100% CO_2_ flush to remove gas bubbles during column commissioning. During experimentation, anoxic conditions were maintained by application of a 99:1 N_2_:CO_2_ gas mixture sparged through the seawater reservoir. VFA preparations were also deoxygenated actively with N_2_. Among 20 columns, 3.3 mM nitrate salts (calcium nitrate salt in the form of Yara Petrocare 45) were applied to 10 columns (for mitigation of biosouring), whereas no nitrate treatment was involved in the remaining columns (Table 1). This experimental setup was established at BP Biosciences Center.

There were three main phases that could be observed in the treated columns, *viz*, sulfidogenesis (S), mitigation/control (M), and rebound sulfidogenesis (R). A transition to the mitigation phase (TM) between sulfidogenesis and mitigation was determined where the sulfide concentration was >1 mM even after the nitrate treatment. Four bioreactors (columns 7, 9, 10, and 14) were operated under anaerobic conditions at ambient lab temperature (∼19°C), and the remaining 16 columns were operated at 30°C (Table 1). The temperature was maintained by water circulation through column jacket assemblies; 30°C was used to replicate conditions supporting mesophilic sulfate-reducing microorganism activity in oil reservoir bioactive zones. Effluent samples were collected from each column over different time points, and microbial diversities of the samples were determined to explore the shift in community structure across different time points and phases (Table 1). Nineteen columns were sacrificed at different time points, and the sessile communities from three different sections (top, middle, and bottom) of the columns were harvested under anaerobic conditions to understand the microbial diversity of the stationary phase (Table 1).

### DNA extraction and sequencing.

For effluent samples, DNA was extracted from 674 filters (covering different columns across different time points) (Table 1) using the MagMAX microbiome ultra-nucleic acid isolation kit, following the manufacturer’s protocol. Next, 96-well standard plates were used for isolation of DNA using the KingFisher Flex system. The MagMAX_Microbiome_Liquid_Buccal_Flex program of the KingFisher Flex system was used for DNA extraction. Three sessile samples (top, middle, and bottom) from each column (a total of 57 samples from 19 columns) were used for DNA extraction. For sessile samples, the whole sections were harvested at a time and resuspended in DNA/RNA Shield to preserve the samples and serve as a lysis buffer for homogenization. Subsequent processing for DNA extraction from sessile samples was conducted using the ZymoBiomics DNA miniprep kit.

Three Illumina MiSeq runs were used to sequence 731 samples (674 effluent samples and 57 sessile samples), and these samples were sequenced to an average depth of 40,709 paired-end reads (SD, 10,527) on the Illumina MiSeq platform. Specifically, the V4 region of the 16S rRNA gene was PCR amplified with 515F-806R primers (20) that included sequencer adapter sequences used in the Illumina flowcell (21). Amplicon library preparations and sequencing were conducted at the Argonne National Laboratory. Amplicon libraries were 2 × 151 paired-end sequenced on the Illumina MiSeq platform.

### Bioinformatic analyses.

Paired-end reads from 731 Illumina MiSeq libraries were filtered, denoised, and merged using DADA2 (22). Samples from three different runs were processed separately in DADA2, considering different error profiles for different runs. The merged reads were inflated to redundant fasta files using deunique_dada2.py (https://github.com/bowmanlab/seq_data_scripts/blob/master/deunique_dada2.py) for analysis using paprica. The output from deunique_dada2.py (.exp.fasta) was analyzed using paprica v0.7.0 (https://github.com/bowmanjeffs/paprica/releases/tag/paprica_v0.7.0) for the determination of the bacterial community and predicted metabolic structure (15). In brief, paprica places each read on a phylogenetic reference tree created from complete 16S rRNA genes from all completed genomes in GenBank. Placements to terminal branches on the reference tree are referred to as closest completed genomes (CCGs), while placements to internal branches are referred to as closest estimated genomes (CEGs). The taxonomy of the unique sequences reported in the manuscript is based on taxonomic affiliations of CCGs or CEGs. The output of the paprica metabolic inference is an estimate of the enzymes and metabolic pathways contained in each member of the community. The paprica pipeline depends on RAxML-ng for reference tree construction (23), and it uses Infernal (24) and EPA-ng (25) for phylogenetic placement. It further makes use of Gappa (26) and Pathway Tools (27). Further analyses were carried with 16S rRNA gene copy number-corrected abundances of unique sequences generated using paprica. Abundances of SSPs were obtained by adding up the abundances of all the unique sequences assigned to a particular closest completed genome of an SRB.

### Cell counts.

Flow cytometry of 553 samples was used to determine the total cell abundances in the effluents. These 553 samples cover effluents from S, TM, M, and R phases of both treated and nontreated columns. A total of 1 ml of effluent samples was collected during the same time points for DNA extractions from all the columns for cell counting using the Guava easyCyte 11HT benchtop flow cytometer. The samples were fixed with 25% glutaraldehyde to a final concentration of 0.25% and prefiltered using 60-μm filters to remove any larger debris. A total of 200 μl of samples was transferred to 96-well plates, where count beads (123count eBeads; Invitrogen) were added to each well for absolute cell counting. The samples were stained with SYBR green before the cells were quantified. Cell abundances were counted from green fluorescence versus forward scatterplots using custom R scripts (https://github.com/bowmanlab/flow_cytometry_scripts). For further analyses, outliers (18 observations) for cell abundances from each phase were determined using Tukey’s method (28) and removed from the data set. An observation was considered an outlier when its value was outside the range [Q1 − 1.5 × (Q3 − Q1), Q3 + 1.5 × (Q3 − Q1)], where Q1 and Q3 are the first and third quartiles, respectively. Cell abundances of SSPs were calculated using relative abundance data of SSPs and total cell count per ml of a sample using equation 1, as follows:

(1) $$\text{cell abundances of SSPs (per ml) = }\frac{\text{relative abundance of SSPs (\%)}}{100}\times \text{total cell count per ml}$$

The relative abundances of SSPs were calculated based on the total abundances of unique bacterial sequences in each sample. The shift in cell abundances for SSPs across phases could likely be improved by a better representation of archaeal community structure.

### Statistical analyses.

All the statistical analyses were carried out in R and R Studio (29). Alpha diversity indices of treated and nontreated columns across different phases were calculated using the phyloseq package (30). Differences in Shannon’s and Simpson’s indexes were analyzed. For the comparison of alpha diversity patterns between sessile and effluent samples, the Wilcoxon test was conducted. The Kruskal-Wallis test for significance was used to understand whether the overall changes in alpha diversity across different phases were significant, whereas the Wilcoxon test was used to find out the pairwise significance for the differences in alpha diversity among different phases. A principal coordinates analysis (PCoA) of the Bray–Curtis dissimilatory based on the abundance of all the bacterial unique sequences across samples from treated columns was performed using phyloseq (30) to understand the shift in microbial diversity across time and phase. PCoA of Bray-Curtis dissimilatory based on the abundances of all the bacterial unique sequences from sessile and three effluent time points of each column (before the columns were sacrificed for sessile community harvesting) was conducted to understand the relationships between sessile and effluent samples at a given time point. A canonical analysis of principal coordinates (CAP) based on Bray-Curtis dissimilatory was done on samples from TM, R, and M phases of treated columns, constraining the top three most abundant SRBs found across the treated columns. Since samples from the S phase were not harvested for treated columns, the effluent S phase was not considered for CAP analysis. All the ordination plots were based on relative abundances (%) of the bacterial unique sequences. Anomalies of the relative abundance of the SSPs were calculated and compared across different time points in the treated columns to get an overall idea of the shift in SSP abundances across different time points. The SSP anomalies were calculated based on equation 2, as follows:

(2) $$\text{anomaly (SSP) = }\frac{\text{SSP relative abundance (\%) }-\text{ column mean of SSP relative abundance (\%)}}{\text{column mean of SSP relative abundance (\%)}}$$

Boxplots were constructed to compare the differences in the cell abundances of the SSPs across different phases. The Kruskal-Wallis test for significance was used to understand whether the overall changes in cell abundance of SSPs across different phases are significant, whereas the Wilcoxon test was used to find out the pairwise significance for the differences in SSP cell abundances between the phases.

The dissimilatory sulfate reduction I (to hydrogen sulfide) (DSR) pathway was selected from the output of paprica for anomaly analyses. Anomalies of the relative abundance of the DSR pathway were calculated and compared across different time points in each column to get an overall idea of the shift in pathway abundances across different time points. The anomaly was calculated based on equation 3, as follows:

(3) $$\text{anomaly (DSR) = }\frac{\text{DSR relative abundance (\%) }-\text{ column mean of DSR relative abundance (\%)}}{\text{column mean of DSR relative abundance (\%)}}$$

The average anomaly was calculated for the treated columns, and a generalized additive model (GAM) was used to understand the average anomaly trend. GAM was constructed using the mgcv package (31).

### Data availability.

The sequence data were submitted to the NCBI Sequence Read Archive (SRA) under BioProject number PRJNA714273.
